# Downregulation of the expression of galanin impairs erectile function in hypoandrogenic rats

**DOI:** 10.1093/sexmed/qfad029

**Published:** 2023-06-20

**Authors:** Piao Yuan, Xiong Li, Wen-ju Xiong, Jun Jiang, Rui Jiang

**Affiliations:** Department of Urology, The Affiliated Hospital of Southwest Medical University, Luzhou, 646000, China; Department of Urology, The Affiliated Hospital of Southwest Medical University, Luzhou, 646000, China; Department of Urology, The Affiliated Hospital of Southwest Medical University, Luzhou, 646000, China; Department of Thyroid Surgery, The Affiliated Hospital of Southwest Medical University, Luzhou, 646000, China; Department of Urology, The Affiliated Hospital of Southwest Medical University, Luzhou, 646000, China; Nephropathy Clinical Medical Research Center of Sichuan Province, The Affiliated Hospital of Southwest Medical University, Luzhou, 646000, China

**Keywords:** testosterone, erectile dysfunction, galanin, eNOS

## Abstract

**Background:**

The relationship between galanin and erectile function under low androgen levels is still unclear.

**Aim:**

To explore whether a low testosterone level damages the erection of a rat by regulating the expression of galanin and GalR in penile cavernous tissue.

**Methods:**

Thirty-six male Sprague-Dawley rats, 8 weeks of age, were randomly grouped as follows (n = 6): control, castration, castration + testosterone replacement, control + transfection, castration + transfection, and castration + empty transfection. At 4 weeks after castration, rats in the transfection group were injected with lentivirus carrying the targeting galanin gene (2 × 10^8^ TU/mL, 10 μL) in the corpus cavernosum. After 1 week of injection, the intracavernosal pressure (ICP), mean arterial blood pressure (MAP), nitric oxide (NO), serum testosterone concentration, galanin, GalR1-3, ROCK1, ROCK2, and p-eNOS/eNOS in the rat penile tissues were evaluated.

**Outcomes:**

ICPmax/MAP and the expression of galanin in the corpus cavernosum in castrated rats were obviously decreased as compared with those in the control rats.

**Results:**

The castrated rats showed remarkably lower ICPmax/MAP, galanin, GalR1-3, p-eNOS/eNOS, and NO content and markedly higher ROCK1 and ROCK2 in penile tissues than the control group (*P* < .05). The transfected rats administrated with LV Gal had obviously higher ICPmax/MAP, p-eNOS/eNOS, and NO content and less ROCK1 and ROCK2 protein expression in the corpus cavernosum when compared with the castration group (*P* < .05).

**Clinical Translation:**

Upregulating the expression of galanin in the penile corpus cavernosum might be a novel method of treating erectile dysfunction caused by a low androgen level.

**Strengths and Limitations:**

The conclusions obtained in the animal experiments need to be confirmed in human data.

**Conclusion:**

The erectile function of hypoandrogen rats might be inhibited by downregulating the level of galanin and GalR1-3, upregulating ROCK1 and ROCK2 levels, and inhibiting the eNOS/NO signaling pathway in penile corpus cavernosum.

## Introduction

Erectile dysfunction (ED) refers to the inability of men to continuously develop penile erection to achieve sexual satisfaction.^1^ Several risk factors are correlated with ED, such as dyslipidemia, hypertension, diabetes, aging, and low testosterone levels.^2^^,^^3^ Among them, decreased testosterone level is a major cause of ED.

NOS/NO/cGMP is one of the major signaling pathways of smooth muscle cell (SMC) relaxation and penile erection.^4^ Impaired NO/cGMP signaling is an important cause of ED.^5^ Testosterone deficiency decreases nitric oxide (NO) levels by inhibiting the expression and bioactivity of NO synthase.^6^ A significant decrease of p-eNOS/eNOS and the quantity of endothelial cells (ECs) and SMCs was found in the castrated rat penile tissues, while ROCK1 and ROCK2 expression was remarkably increased. Supplementation of testosterone increased p-eNOS/eNOS and the content of SMCs and ECs, resulting in enhancing the erection of castrated rats.^7^^,^^8^ However, for patients who need to maintain low androgen status, such as those with prostate cancer, it is very important to find ways to ameliorate their erectile function.

Galanin is a proangiogenic factor consisting of 29 or 30 amino acids that may promote vasodilation.^9-11^ Galanin can increase extracellular cGMP levels by stimulating the NO/cGMP pathway in the ventral hippocampus of the rat.^12^ Meanwhile, the NO/cGMP pathway can increase the expression of galanin in rat dorsal root ganglion.^13^ Galanin plays a role in other diseases, such as pain, depression, anxiety, diabetes, seizures, tumors, and spinal cord diseases.^14-16^ In addition, Galanin/GalRs downstream of the MAPK/MEK/eNOS, PI3K/Akt/eNOS, and ROCK/eNOS signaling pathways are involved in penis erection by regulating smooth muscle contraction and relaxation. Galanin exerts biological effects that rely on 3 receptors (GalR1, GalR2, and GalR3). Activated GalR1 and GalR2 stimulate mitogen-activated protein kinase (MAPK) activity, which may lead to the activation of eNOS by reducing MEK pathway activation.^17-19^ However, in hypertensive mice, activation of p38-MAPK may lead to decreased eNOS activation and NO synthesis and release.^20^ Activated GalR2 inhibits the PI3K/Akt pathway and eNOS phosphorylation, ultimately reducing NO synthesis.^21^^,^^22^ Galanin can decrease the activity of RhoA in rat pheochromocytoma cells (PC12 cells), which leads to decreased expression of ROCK and increased eNOS phosphorylation.^23^^,^^24^ The characteristics of GalR3 are still completely unknown; GalR3 probably activates K^+^ influx by activating ATP-sensitive K^+^ channels and GIRK channels (G protein–regulated inwardly rectifying K^+^ channels).^25^ These results suggest that the galanin/GalR signaling pathway may regulate the NO/cGMP pathway and RhoA/ROCK pathway.

Galanin inhibited the contraction of SMCs in the canine small intestine, and in humans, it strongly inhibited gastrointestinal SMC motility and significantly delayed gastric emptying.^2-27^ However, other studies have shown that galanin induced contraction of SMCs in the rat ileum, colon, and bladder; mouse distal colon; and opossum lower esophageal sphincter.^9^^,^^28^^,^^29^ Galanin induced contraction of the rat myometrium by increasing Ca^2+^ concentration and sensitivity.^30^ This may be related to galanin binding to different receptors in different tissues. Activated GalR1 inhibited Ca^2+^ influx by inhibiting voltage-dependent Ca^2+^ channels in myenteric neurons.^31^ Activation of GalR2 leads to opening of the BK channels (big conductance Ca^2+^-activated K^+^) to elevate intracellular Ca^2+^ by the IP3 pathway.^32^ Ca^2+^ and K^+^ are important ion signals during the contraction and relaxation of SMCs. Low testosterone levels decreased galanin mRNA levels in the bed nucleus of the stria terminalis of male Djungarian hamsters and the mRNA levels of GalR1 and GalR2 in proopiomelanocortin neurons of the arcuate nucleus of male Wistar rats. Testosterone supplementation can prevent the effects of castration on the mRNA of galanin, GalR1, and GalR2.^33^^,^^34^ These outcomes show that testosterone may modulate the expression of galanin and GalR. However, the relationship between galanin and GalR in the penile cavernous tissues of rats and the erectile function in castrated rats has not been reported.

Therefore, this study aimed to answer the following question: does low androgen status affect the erectile function in rats by regulating the galanin signaling pathway in their cavernous tissue? Understanding whether hypoandrogen status affects the erectile function in rats via the galanin/GalR signaling pathway may provide a new direction to treat ED.

## Methods

### Animals of grouping

Experiments done in this study were all approved by the Laboratory Animal Management Committee of Southwest Medical University and carried out in accordance with the *Guide for the Care and Use of Laboratory Animals* (US National Institutes of Health Publication No. 85-23, revised 1996). Thirty-six male Sprague-Dawley rats (Laboratory Animal Center of Southwest Medical University) weighing 180 to 220 g, 8 weeks of age, were randomly grouped as follows (n = 6): control (sham), castration (cast), castration + testosterone replacement (cast + T), control + transfection (sham + LV Gal), castration + transfection (cast + LV Gal), and castration + empty transfection (cast + vector). The bilateral testis and epididymis were excised in the castration group. Only the scrotum was incised and sutured in the rats of the sham group. Testosterone propionate (3 mg/kg) was performed by subcutaneous injection into the testosterone replacement group rats every 2 days after surgery, and rats in the other groups received subcutaneous injection of vegetable oil of the same volume.^35^ At the beginning of 5 weeks after surgery, intracavernous injection of the lentivirus vector carrying the galanin gene (2 × 10^8^ TU/mL, 10 μL) was performed in the transfection group, with the same doses and titers of empty vector injected into the empty transfection group.

### Lentivirus transfection

The lentivirus vector LV Gal (80636-1, contract GOSL0328791; JiKai Gene Company) and negative control virus CON335 were used in this experiment. As previously described,^36^ 1% pentobarbital sodium (40 mg/kg) was used to anesthetize rats. A rubber band was used to wrap the root of the rat penis; then, intracavernous injection of the lentiviral vector (2 × 10^8^ TU/mL, 10 μL) carrying the galanin-specific overexpression gene was performed in transfected rats. The rubber band was removed 3 minutes after injection.^37^ The empty vector was injected into the penile tissue of the transfection group with the same volume and titers of negative control virus.

### Detection of intracavernous pressure and mean arterial pressure

After 6 weeks of castration, the rats were anesthetized again in the same way. Heparin-filled injection needles (24G and 26G) are inserted into the corpus cavernosum and the right common carotid artery, respectively, and intracavernosal pressure (ICP) and mean arterial pressure (MAP) are monitored by a pressure sensor, respectively. Then expose the prostate through a midline abdominal incision of the abdomen. Electrical stimulation of the cavernous nerves adjacent to the prostate was performed with 5 milliseconds of amplitude, 12 Hz of frequency, 3 and 5 V of intensity, and 30 seconds of duration by the BL420 Biological Function Experimental System (Techman Technology Co, Ltd).^38^ The interval between the electrical stimuli in this experiment was 15 minutes.

### Examination of serum testosterone

After the evaluation of the ICP and MAP, arterial blood from the right common carotid was collected, and rats with excessive anesthesia were sacrificed. After centrifugation of blood, serum testosterone was detected by a rat testosterone ELISA kit (Lengton Bioscience Co, Ltd) and microplate reader (BioTek).

### Immunohistochemistry

After removal of the prepuce and urethra, the corpus cavernosum tissue was separated into 4 segments from distal to proximal, which were applied to immunohistochemistry, immunofluorescence staining, Western blot, and NO concentration detection. After the paraffin-embedded corpus cavernosum tissue of the penis was sectioned, it was dewaxed, endogenous peroxidase was removed, antigen was repaired, and the tissue was blocked. The tissue sections were incubated in immunohistochemistry wet boxes at 4 °C overnight with primary antibodies (galanin and GalR2 antibodies, 1:100 [Biorbyt]; GalR1 and GalR3 antibodies, 1:100 [Thermo Fisher Scientific Inc]). On the second day, the horseradish peroxidase–labeled secondary antibody (1:100; Sangon Biotech) was incubated with the sections for 30 minutes. After DAB staining, the mean integral optical density values of the positive expression of the brown-yellow stain granules observed under a microscope were measured by Image-Pro Plus 6.0 (Media Cybernetics Inc).^39^^,^^40^

### Immunofluorescence staining

Frozen tissue was sectioned with a frozen microtome (Leica) and stained with DAPI (diamidino-phenyl-indole) under dark conditions. An antifluorescence quencher was added dropwise; the tissue sections were sealed with glass coverslips; and the cells were observed under a fluorescence microscope (Olympus Corporation). The presence of green fluorescence in frozen sections after excitation with blue light indicated positive transfection. The transfection rate was the proportion of green fluorescence cells to the general count of cells (green fluorescence protein/DAPI × 100%).^39^^,^^40^

### Western blot analysis

Penile tissues were homogenized in liquid nitrogen and lysed with cold RIPA lysis buffer mixed with 1× phosphatase and protease inhibitor (Beyotime Biotechnology Co, Ltd) overnight. The supernatant was centrifugated and collected. The mixture of supernatant and 5× loading buffer was then denatured at 99 °C for 10 minutes for Western blot analysis after use of a bicinchoninic acid protein assay kit (Beyotime Biotechnology Co Ltd) to detect the protein concentration. After electrophoresis, polyvinylidene fluoride membranes were accessible for protein to transfer. The membranes were then blocked with 5% skimmed milk for 2 hours and incubated with primary antibodies overnight (galanin and GalR2, 1:1000 [Biorbyt]; GalR1 and GalR3, 1:500 [Thermo Fisher Scientific Inc]; ROCK1 and ROCK2, 1:100 [Santa Cruz Biotechnology]; eNOS, 1:1000 [Abcam]; p-eNOS, 1:1000 [Cell Signaling Technology]). Next, the membranes were incubated with horseradish peroxidase–labeled secondary antibodies (1:5000; Sangon Biotech) at room temperature for 2 hours. Finally, an electrochemiluminescence solution (Beyotime Biotechnology Co, Ltd) was used to immerse the membranes for exposure by the protein imaging system (Bio-Rad Laboratories Inc).^38^^,^^39^

### Determination of the NO concentration in rat corpus cavernosum

The penile tissues of rat were homogenized into powder by the same method. After the addition of buffer (9 μL of phosphorate-buffered saline for every 1 μg of tissue), the mixture was centrifuged at 10000 g for 10 min at 4°C. The supernatant was collected to measure the NO concentration via a kit (Elabscience Biotechnology Co, Ltd) and microplate reader (BioTek).^40^

### Statistical analysis

Prism version 8.0.2 (GraphPad Software LLC) was used to analyze the data, which were represented as mean and standard deviation (mean ± SD). Data normality and distribution were tested by the Shapiro-Wilk test. One-way analysis of variance and linear correlation analysis were adopted for data analysis. Statistical significance was considered at *P* < .05.

## Results

### General data

**Figure 1:**
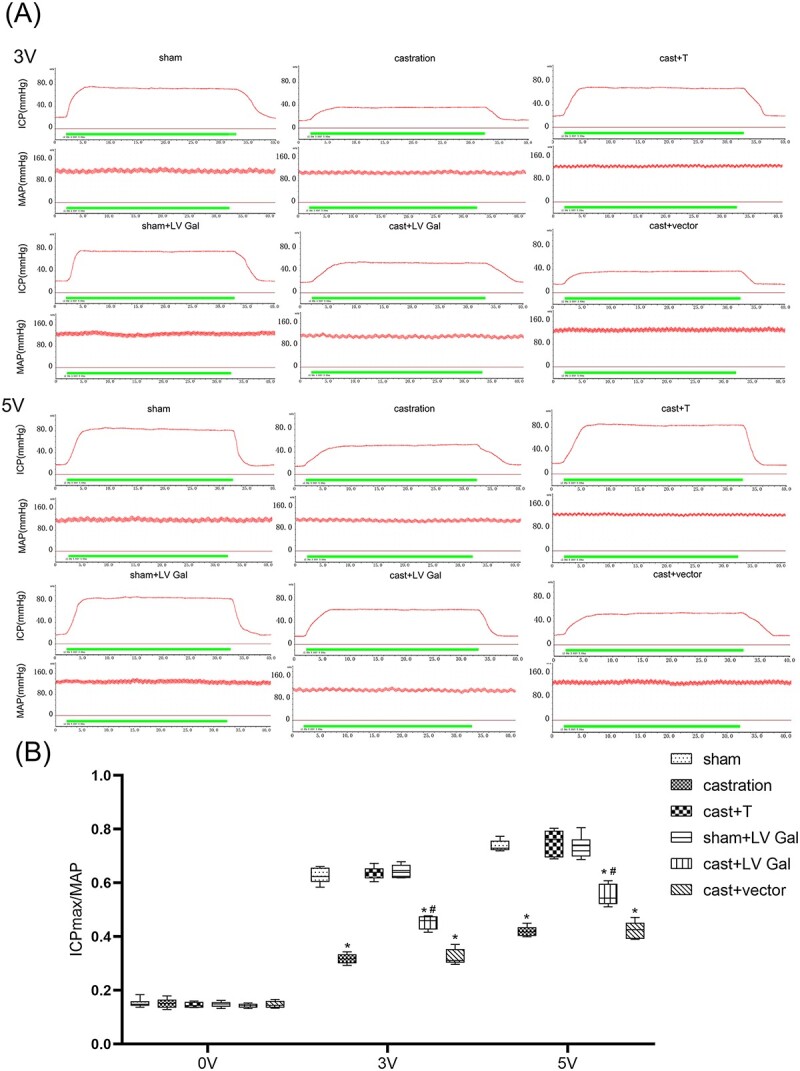
Determination of the ICP and MAP of the penile cavernosum of rats in different groups. (A) Traces of the ICP and MAP electrical stimulations at 3v and 5v among all groups. The ICPmax/MAP was significantly decreased in the castrated rats (0.32 ± 0.018, 3 V; 0.43 ± 0.033,5 V) when compared with thatinthe cast + T group rats (0.64 ± 0.023, 3 V; 0.75 ± 0.048, 5 V) and in the sham group rats (0.63 ± 0.029, 3 V; 0.74 ± 0.02, 5 V). While a significant increase of ICPmax/MAP was evaluated in the transfected rats (0.45 ± 0.025, 3 V; 0.55 ± 0.038, 5 V) in comparison to that in the castrated rats (*P* < 0.01). (B) Data of ICPmax/MAP are represented by box and whisker plots. ^*^P < 0.01 compared with sham and cast + T rat. ^#^*P* < 0.01 compared with cast rat. cast, castration; ICP, intracavernous pressure; MAP, mean arterial pressure; T, testosterone.

Weight and MAP did not differ significantly among the groups. The castrated rats showed deeply declined serum testosterone levels and ICPmax/MAP ratio as compared with the sham and cast + T groups (*P* < .01), while transfected rats had a remarkably higher ICPmax/MAP ratio than the castrated rats (*P* < .01; Figure 1).

### Fluorescence staining in the penile tissues of rats

**Figure 2:**
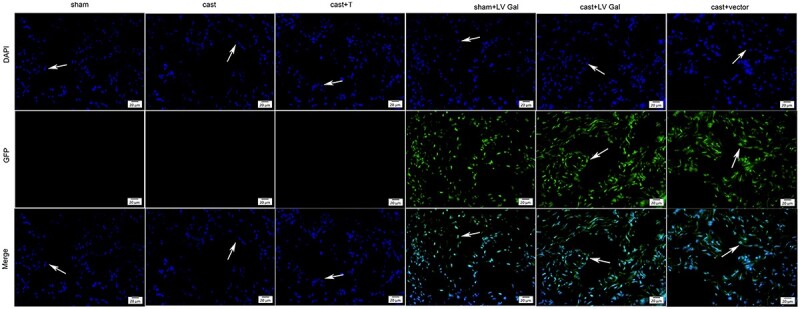
Fluorescence staining in the penile tissues of rats in different groups. Nuclei were stained by DAPI as determined by fluorescence microscopy. Green fluorescence was measured in endothelial cells (white arrows) by lentivirus vector.

Staining was localized to the ECs and some SMCs of the penile cavernous tissues in the sham + LV Gal, cast + LV Gal, and cast + vector groups. The transfection rate was 87.78% ± 3.32% in the sham + LV Gal group, 83.98% ± 5.47% in the cast + LV Gal group, and 85.16% ± 6.97% in the cast + vector group. There was no expression of green fluorescence in the 3 nontransfected groups (Figure 2).

### Galanin and GalR1-3 expression in the penile cavernous tissues of rat by immunohistochemistry

**Figure 3:**
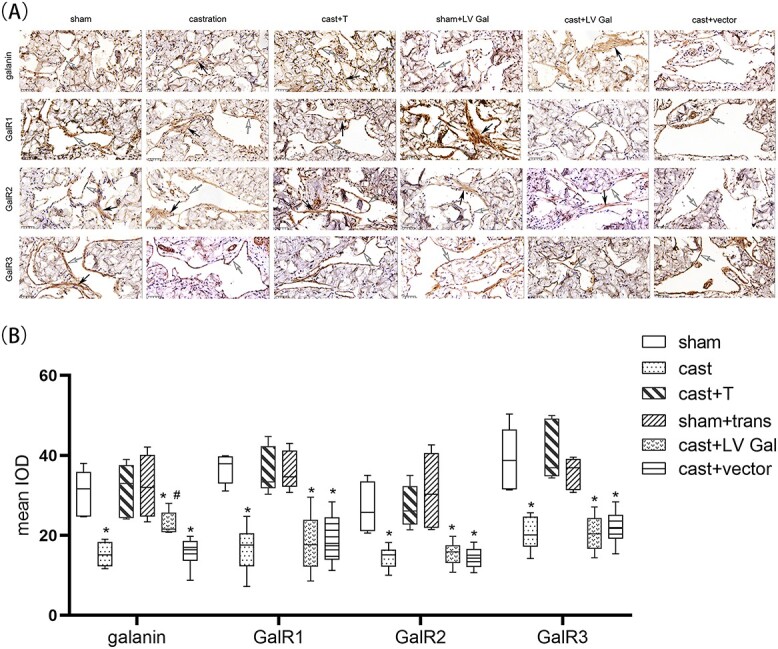
The distribution of galanin and GalR1-3 in the penile cavernous tissues of rats as detected by immunohistochemistry (×40). (A) Galanin and GalR1-3 are respectively located in the membrane and cytoplasm of endothelial cells (white arrows) and smooth muscle cells (black arrows) in the corpus cavernosum tissues of rats. (B) Data are showed as the mean integral optical density of galanin and GalR1-3. ^*^P < .01 vs sham and cast + T rats. ^#^*P* < .01 vs cast rats. cast, castration; T, testosterone.

Galanin and GalR1-3 were respectively distributed in the cytoplasm and membrane of ECs and SMCs in the corpus cavernosum tissues of rat. The castrated rats showed a remarkable decrease of galanin and GalR1-3 levels as compared with the sham and cast + T groups (*P* < .01), while a significant increase in the expression of galanin was noted in the transfected rats in comparison with the castrated rats (*P* < .01; Figure 3).

### Expression of each protein in the penile cavernous tissues of rats by Western blot

**Figure 4:**
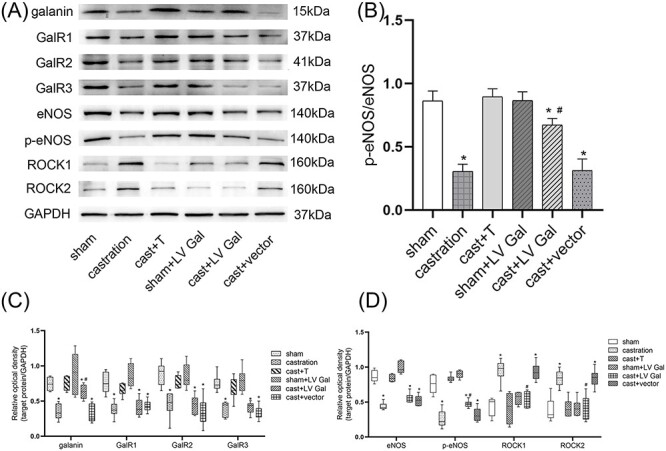
Western blot of the expression of galanin, GalR1-3, ROCK1, ROCK2, eNOS, and p-eNOS in penile cavernous tissues of rats. (A) Western blot for galanin, GalR1-3, ROCK1, ROCK2, eNOS, and p-eNOS in different groups. (B) Data are represented as the p-eNOS/eNOS ratio. (C) Box and whisker plots show the relative density values of galanin, GalR1-3. The expression of galanin, GalR1-3 were significantly reduced in the castrated rats (galanin: 0.35 ± 0.11, GalR1: 0.38 ± 0.11,GalR2: 0.45 ± 0.18,GalR3:0.4 ± 0.11) in contrast with those of the sham (galanin: 0.74 ± 0.1, GalR1:0.76 ± 0.15,GalR2: 0.89 ± 0.16, GalR3: 0.75 ± 0.12) and cast + T groups rats (galanin: 0.75 ± 0.09, GalR1:0.66 ± 0.09, GalR2: 0.77 ± 0.1, GalR3:0.67 ± 0.16). Andan obvious increase of galanin was observed in the transfected rats (galanin: 0.63 ± 0.1, GalR1:0.41 ± 0.12,GalR2: 0.45 ± 0.13,GalR3:0.41 ± 0.08) compared to those of the castrated rats (*P* < 0 .05). (D) Box and whisker plots show the relative density values of ROCK1, ROCK2, eNOS, and p-eNOS. The expression of eNOS, p-eNOS were greatly reduced and ROCK1 and ROCK2 were significantly up-regulated in the castrated rats (eNOS: 0.44 ± 0.05,p-eNOS: 0.25 ± 0.11,ROCK1: 0.95 ± 0.16,ROCK2:0.84 ± 0.12) by comparison with those in the sham (eNOS: 0.87 ± 0.08, p-eNOS: 0.76 ± 0.13,ROCK1: 0.44 ± 0.13,ROCK2:0.38 ± 0.17) and cast + T groups rats (eNOS: 0.84 ± 0.05,p-eNOS: 0.79 ± 0.04,ROCK1: 0.41 ± 0.21.ROCK2:0.41 ± 0.13). And the expression of p-eNOS was significantly increased and a striking decrease of ROCK1 and ROCK2 levels was observedin the transfected rats (eNOS: 0.55 ± 0.07, p-eNOS: 0.48 ± 0.05,ROCK1:0.53 ± 0.11,ROCK2: 0.42 ± 0.17) compared to those of the castrated rats (*P* < 0.05). ^*^P < 0.05 compared with sham and cast + T rat. ^#^*P* < 0.05 compared with cast rat. GalR, galanin receptor; eNOS, endothelial nitric oxide synthase; p-eNOS, phospho-eNOS; ROCK, Rho-associated kinases; cast, castration; T, testosterone.

In contrast with those of the sham and cast + T groups, galanin, GalR1-3, eNOS, and p-eNOS protein expressions and p-eNOS/eNOS ratio were greatly reduced (*P* < .05), and ROCK1 and ROCK2 protein expressions were significantly upregulated in the castrated rats (*P* < .05). The transfected rats administrated with LV Gal had an obvious increase in the expression levels of galanin and p-eNOS and the p-eNOS/eNOS ratio (*P* < .05) and a striking decrease of ROCK1 and ROCK2 levels as compared with the castrated rats (*P* < .05; Figure 4). There was a positive correlation between serum testosterone level and the expression of galanin (*Y* = 0.4033 + 0.01969*x*, *r* = 0.7043), GalR1 (*Y* = 0.3808 + 0.01886*x*, *r* = 0.8031), GalR2 (*Y* = 0.3815 + 0.02272*x*, *r* = 0.8090), and GalR3 (*Y* = 0.3496 + 0.01960*x*, *r* = 0.8382) in the corpus cavernosum.

### NO concentration in rat corpus cavernosum

The castrated rats showed considerably less NO concentration than the sham and cast + T groups (*P* < .01). The concentration of NO in the rats transfected with LV Gal was markedly increased when compared with that in the castrated rats (*P* < .01; Table 1).

**Table 1:** Body weight, MAP, serum testosterone, NO content, and transfection rate in the penile cavernous tissues of rat among all groups.^a^

**Group (n = 6)**	**Body weight, g**	**MAP, mm Hg**	**Testosterone, nmol/L**	**NO, μmol/gprot**
Sham	316.78 ± 6.35	119.60 ± 4.95	20.37 ± 0.96	13.90 ± 0.87
Cast	320.72 ± 9.50	119.10 ± 5.90	1.44 ± 0.37^b^	4.96 ± 0.61^b^
Cast + T	321.63 ± 5.81	118.34 ± 5.82	19.37 ± 1.6	14.28 ± 0.94
Sham + LV Gal	323.85 ± 4.27	118.43 ± 5.39	20.22 ± 0.19	15.66 ± 2.34
Cast + LV Gal	319.70 ± 5.77	119.75 ± 4.74	1.59 ± 0.2^b^	12.75 ± 3.27^b^^,^^c^
Cast + vector	323.10 ± 5.30	117.62 ± 5.87	1.32 ± 0.31^b^	6.86 ± 3.17^b^

Abbreviations: cast, castration; Gal, galanin; LV, lentivirus vector; MAP, mean arterial pressure; NO, nitric oxide; T, testosterone.

a Data are presented as mean ± SD.

b *P* < .01 vs sham and cast + T rats.

c *P* < .01 vs cast rats.

## Discussion

In this study, sham and cast + T groups demonstrated normal NO content and ICPmax/MAP (3 and 5 V), whereas the castration consistently impaired the erectile response. This result showed that a low testosterone level inhibited the erection of rats. After testosterone supplementation, NO content and ICPmax/MAP strikingly increased, improving the erectile function of the cast + T group. These outcomes revealed no difference with previous studies.^35^^,^^41^

This study is the first to report that galanin and GalR1-3 are located in the membrane and cytoplasm of ECs and SMCs in the penile tissue. An obvious decrease of galanin and GalR1-3 expression, ICPmax/MAP and p-eNOS/eNOS ratios, and NO concentration and a remarkable increase of ROCK1 and ROCK2 levels in the corpus cavernosum of castrated rats were detected by comparison with the sham and testosterone replacement rats. There was a positive correlation between serum testosterone levels and the expression of galanin and GalR1-3. These outcomes suggested that galanin and GalR1-3 protein expression decreased in the corpora cavernosum tissue of castrated rats, resulting in an increase in ROCK1 and ROCK2 expression in the corpora cavernosum and a decrease in ICPmax/MAP. Previous studies reported that the upregulation of ROCK in the corpus cavernosum of castrated rats, diabetes rats, cavernous nerve injury rats, and spontaneously hypertensive rats could cause fibrosis and transdifferentiation of SMCs into myoblasts, resulting in cavernous fibrosis and eventually inhibited erectile function.^8^^,^^42-48^ Furthermore, the decrease in galanin and GalR1-3 levels resulted in decreases in p-eNOS/eNOS, NO synthesis and release, and ICPmax/MAP.^8^^,^^42-46^ Testosterone replacement resulted in significant upregulation of galanin and GalR1-3, a decrease of ROCK1 and ROCK2 protein expression in rat penile tissue, an increase of p-eNOS/eNOS and NO release, and eventual improvement of ICPmax/MAP in castrated rats. Some studies found that crosstalk between the RhoA/ROCK signaling pathway and eNOS/NO signaling pathway is involved in the inhibition of eNOS phosphorylation.^24^ Therefore, whether galanin and its receptors directly or indirectly regulate the eNOS/NO signaling pathway in rat corpora cavernosum tissues with low testosterone levels needs further study.

When compared with that of the castrated rats, a partial but obvious enhancement of erectile response was measured in the transfected rats, as shown by remarkably higher ICPmax/MAP ratio, galanin level, p-eNOS/eNOS ratio, and NO concentration. A striking decrease in ROCK1 and ROCK2 levels of the transfected rat were detected via intracavernous injection of LV Gal. These outcomes indicate that galanin upregulation inhibited the upregulated ROCK1 and ROCK2 expression and increased p-eNOS/eNOS and NO content in penile tissues, resulting in increased ICPmax/MAP in castrated rats. However, no remarkable differences in the expression of GalR1-3 were measured in the transfection group. These results suggest that galanin upregulation did not affect the level of GalR1-3 in the rat penile tissues with low androgen status. Moreover, after galanin was upregulated, the downstream effect of the Gal/GalR signaling pathway was upregulated through downregulation of GalR1-3 expression. The role of galanin in the Gal/GalR signaling pathway may be more important than GalR.

Compared with the sham and cast+T groups, there were no significant changes in the expression levels of galanin, GalR1-3, ROCK1, and ROCK2, as well as the p-eNOS/eNOS and ICPmax/MAP ratios and NO content levels in the sham+transfected group. This suggests that lentivirus vectors carrying the galanin-specific overexpression gene may not affect the expression of galanin in rat penile cavernous tissue after transfected into normal androgen-state rat penile cavernous tissue.

In this study, the downregulation of galanin and GalR1-3 expression and the subsequent upregulation of the RhoA/ROCK pathway and downregulation of the eNOS/NO pathway may be one of the causes of the decrease in ICPmax/MAP in castrated rats. Specific LV Gal lentiviruses upregulated the level of galanin in the hypoandrogen rat penile tissues, inhibited the upregulation of ROCK1 and ROCK2, increased the p-eNOS/eNOS ratio, and promoted NO release, thus improving ICPmax/MAP. Therefore, upregulation of galanin may be a therapeutic direction for ED induced by low testosterone levels. These results provide a new insight to the research and treatment of ED caused by hypoandrogen status. However, they did not explain the specific role of GalR and which GalR subtype plays a major role by binding to galanin in the processes of erection, thus requiring further study. Meanwhile, the conclusions obtained in the animal experiments need to be confirmed in human data. In addition, the Gal/GalR signaling pathway was shown to activate eNOS phosphorylation through the MAPK/MEK signaling pathway in a previous study.^17-19^ The relationship between Gal/GalR and the MAPK/MEK pathway in the hypoandrogen state needs further studies. A costaining of galanin and galanin receptors with smooth muscle markers or endothelial markers could provide more insight into galanin functional mechanisms. In sum, it is necessary to clarify the role and status of the galanin signaling pathway in the penile erection signaling network.

## Author contributions

P.Y., X.L., J.J., and R.J. participated in the design of the trial and conducted the data acquisition. P.Y., X.L., J.J., and R.J. drafted and revised the manuscript. P.Y., X.L., W.X., and R.J. interpreted and analyzed the data. P.Y., X.L., and W.X. guided the experiment directions and revised the manuscript. P.Y. performed the statistical analysis. All authors read and approved the final version of the manuscript.

## Funding

This study was supported by the Research Foundation of the Department of Human Resources and Social Security of Sichuan Province of Returned Scholars (grant 2019-76).

*Conflicts of interest:* The authors declare that the research was conducted in the absence of any commercial or financial relationships that could be construed as a potential conflict of interest.

## Supplementary Material

supplement_data_qfad029
