# Supplementary material for: Downregulation of the expression of galanin impairs erectile function in hypoandrogenic rats
Source: Sex Med. 2023 Jun 20;11(3):qfad029. doi: 10.1093/sexmed/qfad029 (PMC10281959; doi:10.1093/sexmed/qfad029)
Supplement: supplement_data_qfad029 [file supplement_data_qfad029.doc]

The transfection rate was 87.78±3.32% in the sham + LV Gal group, 83.98±5.47% in the cast + LV Gal group, and 85.16±6.97% in the cast + vector group. There was no expression of green fluorescence in the three non-transfected groups.
